# Estimating the incubation period of hand, foot and mouth disease for children in different age groups

**DOI:** 10.1038/s41598-017-16705-7

**Published:** 2017-11-28

**Authors:** Zhongzhou Yang, Qiqi Zhang, Benjamin J. Cowling, Eric H. Y. Lau

**Affiliations:** WHO Collaborating Centre for Infectious Disease Epidemiology and Control, School of Public Health, Li Ka Shing Faculty of Medicine, The University of Hong Kong, Hong Kong Special Administrative Region, China

## Abstract

Hand, foot and mouth disease (HFMD) is a childhood disease causing large outbreaks frequently in Asia and occasionally in Europe and the US. The incubation period of HFMD was typically described as about 3–7 days but empirical evidence is lacking. In this study, we estimated the incubation period of HFMD from school outbreaks in Hong Kong, utilizing information on symptom onset and sick absence dates of students diagnosed with HFMD. A total of 99 HFMD cases from 12 schools were selected for analysis. We fitted parametric models accounting for interval censoring. Based on the best-fitted distributions, the estimated median incubation periods were 4.4 (95% CI 3.8–5.1) days, 4.7 (95% CI 4.5–5.1) days and 5.7 (95% CI 4.6–7.0) days for children in kindergartens, primary schools and secondary schools respectively. From the fitted distribution, the estimated incubation periods can be longer than 10 days for 8.8% and 23.2% of the HFMD cases in kindergarten and secondary schools respectively. Our results show that the incubation period of HFMD for secondary schools students can be longer than the ranges commonly described. An extended period of enhanced personal hygiene practice and disinfection of the environment may be needed to control outbreaks.

## Introduction

Hand, foot and mouth disease is a childhood disease caused by various viruses that belong to the enterovirus genus including coxsackieviruses, echoviruses and enteroviruses^1^, characterized by fever and vesicular sores on the hands, feet and mouth^2^. Large outbreaks of HFMD have occurred frequently in Asia^3^ and occasionally in Europe^4^ and the US^5^ mainly affecting children under 5 years old^6^. Severe complications may sometimes arise with neurological, cardiovascular or respiratory problems^7^. HFMD causes an average of 500–900 deaths each year in China^3^. The incubation period, defined as the time from infection to symptom onset, is one of the important parameters to guide disease control and prevention^8^.

The incubation period of HFMD is typically described as about 3–7 days^9^. However a recent and an earlier systematic reviews highlighted a lack of empirical evidence for the incubation period of HFMD^10,11^. For example, one study reported that the incubation period of HFMD is ‘usually 3–4 days, but can be as long as 10 days or more’^12^, while in another study the incubation period was described as ‘ranging from 5 to 7 days’^13^. However, evidence supporting these ranges of incubation period was not provided, and from these descriptions it is difficult to determine the proportion of HFMD patients with incubation periods falling within these ranges. Furthermore, incubation period can be varied by age^14^ but this has not been investigated for HFMD.

In 2015–2016, there were unexpected HFMD outbreaks in secondary schools in Hong Kong. This provides data from a wider age range of HFMD cases. In our study, we estimated the incubation period of HFMD for children in different age groups from school outbreaks in Hong Kong.

## Results

We recruited a total of 1458 participants from 97 classes in 35 schools (24 kindergartens, 3 primary and 8 secondary schools), including 309 clinically diagnosed HFMD cases. After selecting classes with ≥2 cases for further analysis, a total of 65 classes with 286 HFMD cases were identified. We further removed 147 cases with missing onset or absence dates and the subsequent cases in the same class and 40 isolated cases. A total of 99 HFMD cases were available for analysis from 12 schools (4 kindergartens, 1 primary and 7 secondary schools). We identified 1 case with prior household infection and was considered not to be infected in class, but possible to infect other classmates.

Table 1 shows the basic characteristics of the HFMD cases in our study. Among the 99 HFMD cases, 64.5% are males. The mean age was 12.1 years (standard deviation 4.7 years). The median time from symptom onset to sick absence was 2 days (interquartile range (IQR) 0–2 days) and the median number of HFMD cases per class was 2 (IQR 2–4 cases). The median days from the onset dates of the first to the last cases per class was 7.5 (IQR 5–14 days). On average, there were 1.3 infectors (range: 1–5) associated with each symptom onset to define the exposure period.

**Table 1:** Characteristics of the 99 Diagnosed Cases of Hand, Foot and Mouth Disease From School Outbreaks in Hong Kong, 2015–2016.

Characteristics	N = 99	(%)
Male	64	64.6%
Mean age, years (SD)	12.1	4.7
School type		
Kindergarten	17	17.2%
Primary school	9	9.1%
Secondary school	73	73.7%
Median time from symptom onset to sick absence, days (IQR)	2	0–2

**Figure 1:**
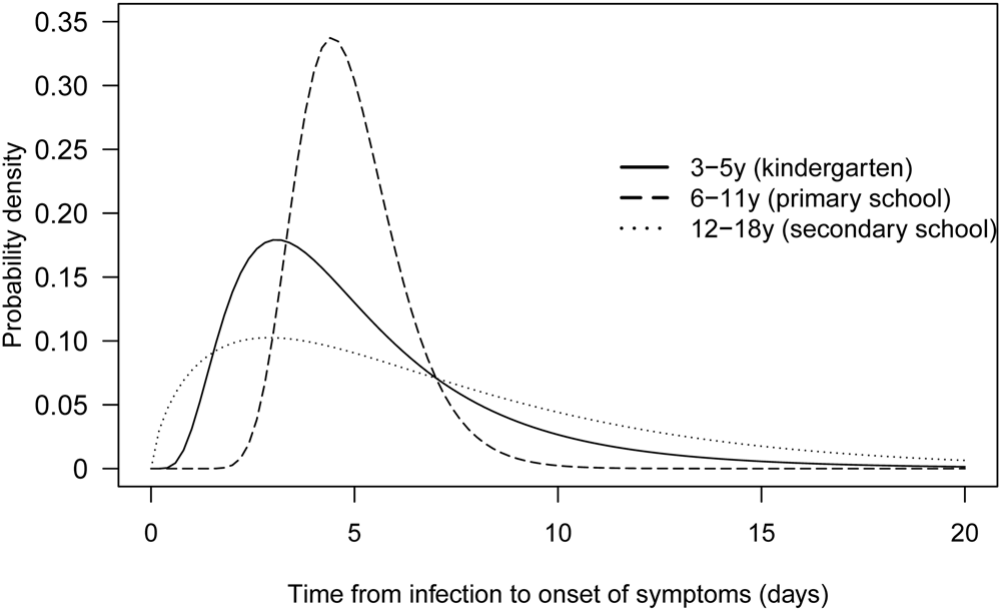
Best-fitted distributions for the incubation period of HFMD. Estimation was based on 17, 9 and 73 selected HFMD cases from outbreaks in kindergartens, primary schools and secondary schools respectively in Hong Kong, 2015–2016. The best fitted distributions were log-normal for kindergartens and primary schools, and gamma for secondary schools.

Based on the symptom onset dates and sickness absence dates of the HFMD cases included for analysis, we fitted log-normal, gamma and Weibull distributions to the observed ranges of the incubation periods (Table 2). Gamma was the best-fitted model with the lowest AIC for secondary and all schools, and log-normal was the best fitted distribution for kindergartens and primary schools. Other distributions also fitted well with AIC differences <3 comparing to the best-fitted distribution. Most of the distributions have satisfactory fit to the observed data, except for primary school where we had a small sample size (see Supplementary Fig. S2). The estimated median incubation period for all children from the Gamma distribution was 5.4 (95% CI 4.4–6.5) days, with estimated 5% and 95% percentiles of 1.0 and 16.0 days respectively (Fig. 1). The incubation period for kindergarten students were shortest with a median of 4.4 (95% CI 3.8–5.1), while that for secondary school students were longest with a median of 5.7 (95% CI 4.6–7.0). However the differences were not statistically significant (bootstrap tests, all p-values >0.07).

**Table 2:** Estimated incubation period for HFMD from different age groups.

School type (age range)	Median (95% CI)/AIC		
	Weibull	Gamma	Log-normal
Overall (2–18 y)	5.5 (4.5–6.7)	5.4 (4.4–6.5)	4.9 (4.0–5.9)
	243.9	242.4	242.5
Kindergarten (2–5 y)	4.6 (3.9–5.5)	4.7 (4.0–5.5)	4.4 (3.8–5.1)
	31.1	29.9	28.3
Primary school (6–12 y)	4.7 (4.4–5.1)	4.8 (4.4–5.1)	4.7 (4.5–5.1)
	23.9	23.2	23.0
Secondary school (12–18 y)	5.9 (4.8–7.2)	5.7 (4.6–7.0)	5.1 (4.2–6.3)
	192.1	191.7	193.6

As a sensitivity analysis, we included the first and at most three subsequent cases in each class, in which 42 HFMD cases were included for the analysis. The estimated median incubation period for all children was then 5.7 (95% CI: 4.7–6.8) days, with 5% and 95% percentiles of 1.3 and 15.2 days respectively. For the sensitivity analysis which restricted to single class outbreaks in each grade, 34 HFMD cases were included for the analysis. The estimated incubation period was 4.8 (95% CI 3.8–5.8) days, with 5% and 95% percentiles of 0.9 and 14.0 days respectively.

## Discussion

We estimated the median incubation period of HFMD to be 4.4 (95% CI 3.8–5.1) days for kindergarten students aged about 2–5 years, consistent with the range of about 3–7 days commonly cited by different health agencies^10^. The estimated median incubation period of HFMD was 5.7 (95% CI 4.6–7.0) for secondary school students aged about 12–18 years and appears to be longer, though the difference did not reach statistical significance with the small sample size in the study. The estimated incubation periods can be longer than 10 days for 8.8% and 23.2% of the kindergarten and secondary school students respectively, based on the fitted distribution. The estimated incubation period for primary school children (n = 9) had a relatively low variability when comparing to other groups, probably because the data were obtained from a single school and hence likely to be caused by a single causative pathogen. In contrast, the relatively high variability of the estimated incubation period in the secondary school students (n = 73) may be explained by higher heterogeneity in host immune responses due to more previous exposure to different pathogens.

To our knowledge, this is the first study to estimate the incubation period distributions of HFMD for different age groups based on empirical data. While the infection times were usually unobservable, the relatively short period between illness onset and sick absence in the school setting has helped reducing the uncertainty in the exposure period. The estimated incubation period was not sensitive to uncertainty due to unobserved transmission chain in outbreaks which may involve multiple generations. Our finding may have implications on the intervention or management of outbreaks in school settings, such as defining evidence-based observation period of HFMD outbreaks and maintaining a longer period of enhanced personal hygiene practice and disinfection of the environment^11^.

Our study has some caveats. There could be recall bias on the dates of symptom onset and sick absence. In the questionnaire, we included individual questions on onset dates of the main symptoms of HFMD, and the start and end dates of sick absence. By prompting the participants to report the sequence of events related to the HFMD episode, the responses tended to be more coherent. We could not rule out potential infections from the community. However, we observed limited transmission from household in our study and the probability of community transmission should be even smaller during the limited period of school outbreaks. Finally, we did not collect specimens for laboratory testing, and therefore we lacked information on the causative virus strain and also may have misclassified HFMD cases due to the variation in the symptoms. During the study period, the main circulating strain in the community was Coxsackievirus A6 which associated with 58% of the outbreaks, followed by Enterovirus 71^15^. Further study is needed to assess potential heterogeneity in the incubation period for different virus strains. With the exception of vaccination against Enterovirus 71, other public health preventive measures against HFMD usually do not consider differences in serotypes. Our results could improve the evidence base for the control of HFMD.

## Materials and Methods

### Sources of data

We recruited kindergartens, primary and secondary schools in February–May, 2015 and September 2015–January 2016, covering the major peak HFMD seasons in Hong Kong. We recruited all students from the classes which reported HFMD cases. Classes with ≥2 HFMD cases were selected for the present analysis of incubation periods.

We distributed questionnaires to the parents or legal guardians of the participated students to collect demographic and epidemiological information. We also collected key information related to disease transmission and progression of HFMD, such as dates of symptom onset (specifically for fever, oral ulcer and rash), dates of sick absence, and potential epidemiological link to other HFMD cases in household. Incentives were provided for the HFMD cases who provided more detailed information. We also collected attendance records from the schools, stratified by HFMD cases and non-cases to assess data completeness.

### Ethics approval

Written informed consent was provided by all participating schools. Proxy written consent from parents or legal guardians were obtained for the students. The study protocol was approved by the Institutional Review Board of the University of Hong Kong/Hospital Authority Hong Kong West Cluster and was performed in accordance to relevant guidelines.

### Statistical analysis

We fitted parametric distributions to the time from infection to the earliest symptom onset of fever, oral ulcer or rash^16^. The exact infection times were not observable but the exposure windows were defined from the symptom onset to the day before sick absence, assuming HFMD cases were infectious only after symptom onset^17^. All potential infectors in the same class were included to construct the exposure windows, assuming limited transmission between different classes. Cases with missing illness onset or absence date, along with subsequent cases in the same class were excluded from the analysis. In other words, only transmissions with complete observation of exposure windows were included in the analysis. Students with earlier HFMD cases in their households were assumed to be infected outside schools, and were included in the analysis only for the exposure to subsequent cases. We also accounted for weekends and school holidays during which there were no exposure in schools. We used maximum likelihood to fit gamma, Weibull and log-normal distributions to the potential incubation intervals and selected the best models using Akaike information criterion (AIC). We estimated the median incubation period for children in kindergartens (aged about 2–5 years), primary (aged about 6–11 years) and secondary schools (aged about 12–18 years) respectively and computed the relevant 95% confidence interval (CI) by the parametric bootstrap approach using 1000 replicates. The 95% percentile of the incubation period was then calculated from the fitted distribution^16^. Further details of the statistical analyses are described in the Supplementary Information.

### Sensitivity analyses

To examine the potential impact of using outbreak data for estimating the incubation period distribution, we analyzed a subset of the cases which conservatively included the primary cases and at most three subsequent HFMD cases in each class. Considering a reproductive number of 2.5–5.5 for HFMD transmission in Hong Kong^18^, this should include most of the first generation cases. To assess sensitivity of the result to the assumption of limited inter-class transmission, we identified school grades with reported HFMD cases in a single class only and estimated the incubation period distribution in this subset. All analyses were conducted by R version 3.3.2. (R Foundation for Statistical Computing, Vienna, Austria).

## Electronic supplementary material

Supplementary appendix
