# Supplementary material for: Estimating the incubation period of hand, foot and mouth disease for children in different age groups
Source: Sci Rep. 2017 Nov 28;7:16464. doi: 10.1038/s41598-017-16705-7 (PMC5705633; doi:10.1038/s41598-017-16705-7)
Supplement: Supplementary file 1 — Supplementary appendix [file 41598_2017_16705_MOESM1_ESM.pdf]

SUPPLEMENTARY APPENDIX

Estimating the incubation period of hand, foot and mouth disease for children in different age groups

Zhongzhou Yang[1], Qiqi Zhang[1], Benjamin J. Cowling[1], Eric H. Y. Lau[1*]

[1]WHO Collaborating Centre for Infectious Disease Epidemiology and Control, School of Public Health, Li Ka Shing Faculty of Medicine, The University of Hong Kong, Hong Kong Special Administrative Region, China.

*Corresponding author: ehylau@hku.hk

The objective of the study is to estimate the incubation period of hand, foot and mouth disease (HFMD). We collected symptom onset date, sick absence date and household HFMD infection from HFMD cases in school outbreaks, which provided information on the potential exposure period. Allowing for school days and holidays, we estimated and selected the best statistical distribution to describe the incubation period for HFMD.

Data description

We collected key information related to disease transmission and progression of HFMD, such as dates of symptom onset (specifically for fever, oral ulcer and rash), dates of sick absence for the diagnosed HFMD cases, and potential epidemiological link to other HFMD cases in household. The symptom onset date of a HFMD case was defined by the earliest onset date of fever, oral ulcer or rash. We selected 12 schools with 30 classes and a total of 99 cases (17, 9 and 73 cases from kindergartens, primary and secondary schools respectively) for the analysis.

Study assumption

We assumed HFMD transmission occurred mainly within class. Inter-class transmission was assumed to be negligible. We also assumed sick leave was taken in the morning, so a HFMD case was absent from school for the whole day of sick leave. Hence, if a HFMD case reported identical symptom onset and absent date, other classmates in the same class will not be exposed to the virus shed by this case.

Identification of the primary cases

A primary HFMD case was identified from each class who had the earliest symptom onset date. In case there were multiple primary cases in one class, we assumed these primary cases as multiple introductions to the class who did not infect each other. In our dataset, only 3 classes were found to have multiple primary cases (n=6). On the other hand, an early case were regarded as an isolated case and excluded from the analysis if no subsequent case was identified in 10 days following the symptom onset date. In our dataset, 4 cases were regarded as isolated primary cases.

Interval censoring

The exact times of infection and symptom onset were unobserved but interval censored. For a HFMD case, the possible time of exposure to subsequent cases in the same class was defined from the symptom onset day to the school day before taking sick leave (Figure S2). The time differences between the exposure period to all potential infectors and the symptom onset day of the subsequent HFMD cases were used to estimate the incubation period.

We defined L_i and R_i as the minimum and maximum possible incubation period respectively. Accordingly the incubation period was interval censored by (L_i, R_i). We allowed a half-day exposure in the daytime to account for the normal school period, while symptom onset may occur any time during the reported symptom onset day. A half-day adjustment was hence applied to the calculation of L_i. Specifically, L_i and R_i were calculated as below:

$$L_i = D'_o - D_A + 0.5$$

$$R_i = D'_o - D_o + 1$$

where D'_o is the symptom onset day for case *i*; D_A, D_o are the first absent day and symptom onset days, respectively, among the potential infectors of case *i*. Similarly, we also accounted for weekends and holidays where there was no contact in schools. This may form m_i disjoint intervals for the possible incubation period (L_{ij}, R_{ij}), j = 1,2,3……m_i for case *i*. The exposure period L_{ij} and R_{ij} are given by:

$$L_{ij} = D'_o - \left(WH_{start,j} - 1\right) + 0.5$$

$$R_{ij} = D'_o - \left(WH_{end,j} + 1\right) + 1$$

Parameter estimation

We assumed several possible distributions for the incubation period, such as log-normal, gamma and Weibull distributions. Accounting for interval censoring, the likelihood is given by:

$$L = \prod_{i=1}^{n}\sum_{j=1}^{m_i}\{F(R_{ij}) - F(L_{ij})\}$$

Where F is the cumulative distribution function for the assumed incubation distribution. The parameters for the distribution were estimated using maximum likelihood method.

Figure S1. The fitting of the three candidate distributions to observed incubation period, stratified by school type. The histogram display the approximate distribution of the incubation period (interval-censored) by showing the empirical distribution of the mid-point (gray) and the full interval (black) of the interval-censored incubation periods.

Figure S2. Possible range for the incubation period of the infectee, defined by symptom onset of the infector and the absent date of the infectee.

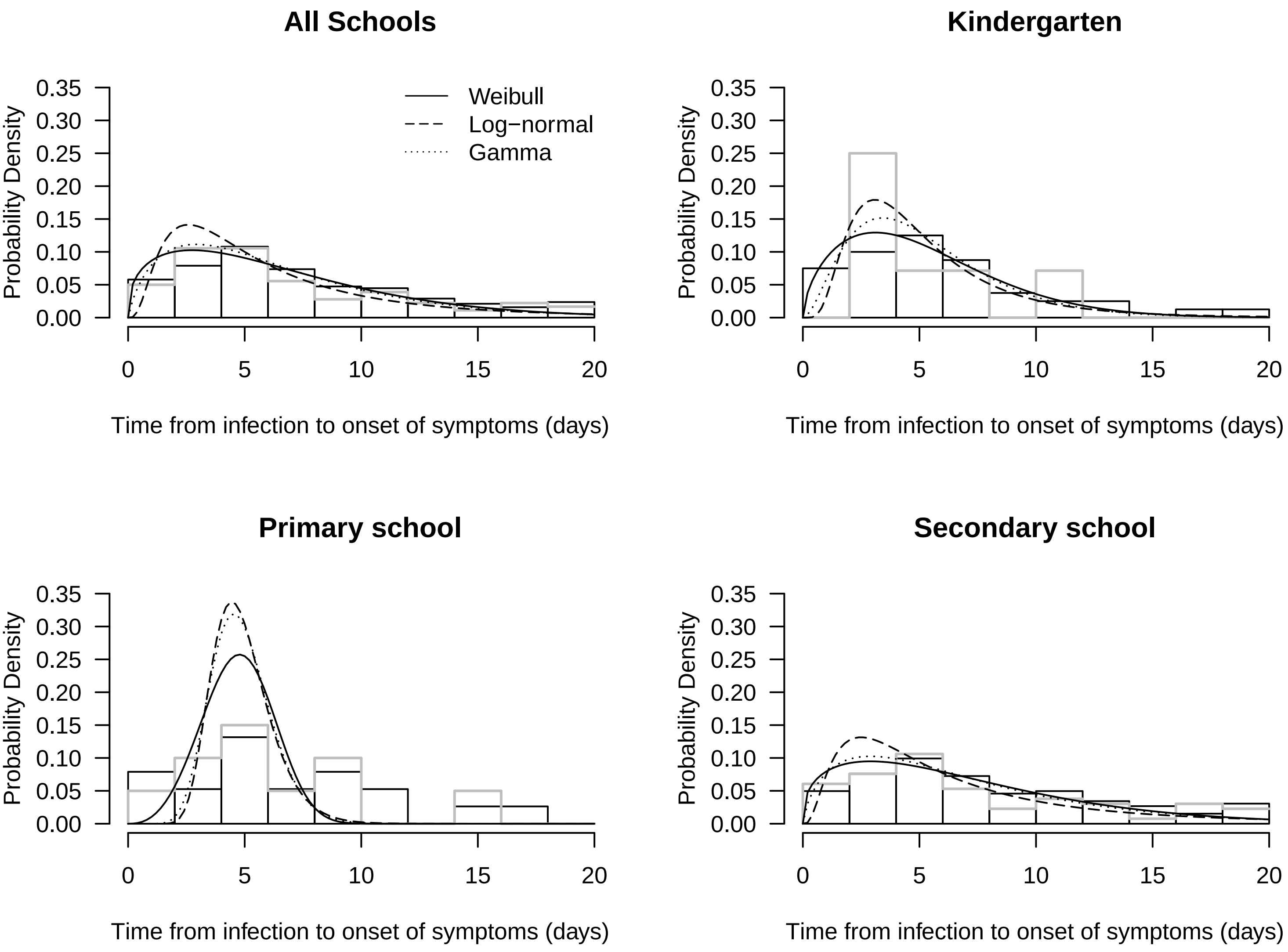
All Schools
Kindergarten
Primary school
Secondary school
Weibull
Log−normal
Gamma
Probability Density
Time from infection to onset of symptoms (days)
0.00
0.05
0.10
0.15
0.20
0.25
0.30
0.35
0
5
10
15
20

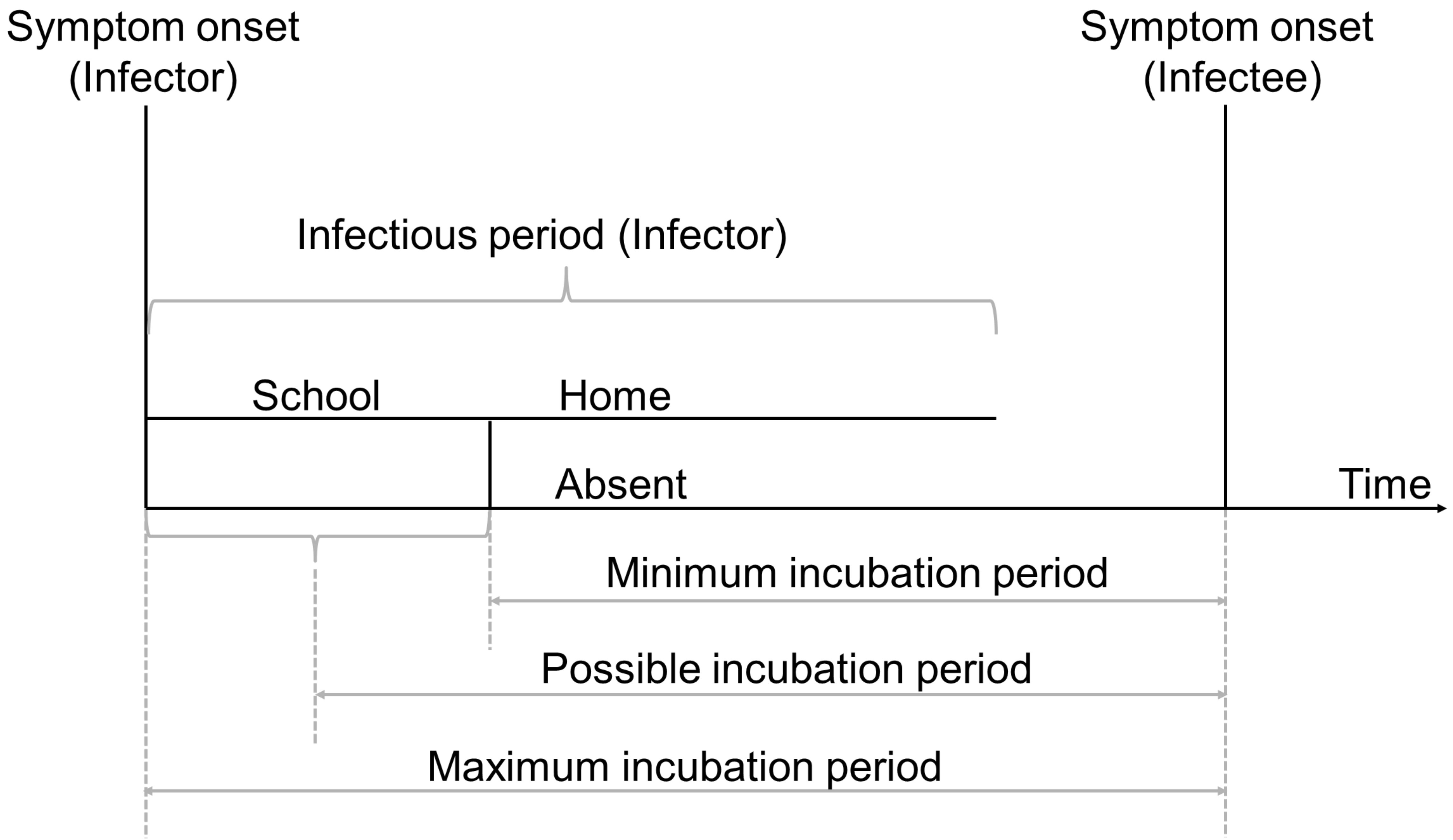
Symptom onset
(Infector)
Symptom onset
(Infectee)
Infectious period (Infector)
School
Home
Absent
Time
Minimum incubation period
Possible incubation period
Maximum incubation period
